# Accelerating development of engineered T cell therapies in the EU: current regulatory framework for studying multiple product versions and T2EVOLVE recommendations

**DOI:** 10.3389/fimmu.2023.1280826

**Published:** 2023-11-24

**Authors:** Delphine Ammar, Inga Schapitz, Maik Luu, Michael Hudecek, Miriam Meyer, Timmothy Taps, Bernd Schröder, Zoltán Ivics, Carmen Sanges, Paul Franz, Ulrike Koehl, Helene Negre, Inez Johanna, Jacquelyn Awigena-Cook

**Affiliations:** ^1^ Regulatory Affairs, Astellas Pharma B.V., Leiden, Netherlands; ^2^ Regulatory Affairs, Bayer Vital GmbH, Leverkusen, Germany; ^3^ Lehrstuhl für Zelluläre Immuntherapie, Medizinische Klinik und Poliklinik II, Universitätsklinikum Würzburg, Würzburg, Germany; ^4^ Regulatory Affairs, Immatics Biotechnologies GmbH, Tuebingen, Germany; ^5^ Regulatory Affairs, Century Therapeutics Inc., Philadelphia, PA, United States; ^6^ Regulatory Affairs, Miltenyi Biotec B.V. & Co. KG, Bergisch Gladbach, Germany; ^7^ Research/Division of Hematology, Gene and Cell Therapy, Paul Ehrlich Institute, Langen, Germany; ^8^ Department of Cell and Gene Therapy Development, Fraunhofer Institute for Cell Therapy and Immunology, Leipzig, Germany; ^9^ Institute of Clinical Immunology, University of Leipzig, Leipzig, Germany; ^10^ Institut de Recherches Internationales Servier, Gif-sur-Yvette, France; ^11^ Department of Hematology and Innovation Center for Advanced Therapy (ICAT), Universitair Medisch Centrum (UMC) Utrecht, Utrecht, Netherlands; ^12^ Global Regulatory Sciences, Bristol Myers Squibb, Uxbridge, United Kingdom

**Keywords:** advanced therapy medicinal products (ATMP), engineered T cell therapies, clinical development, multiple product candidates, parent-child approach

## Abstract

To accelerate the development of Advanced Therapy Medicinal Products (ATMPs) for patients suffering from life-threatening cancer with limited therapeutic options, regulatory approaches need to be constantly reviewed, evaluated and adjusted, as necessary. This includes utilizing science and risk-based approaches to mitigate and balance potential risks associated with early clinical research and a more flexible manufacturing paradigm. In this paper, T2EVOLVE an Innovative Medicine Initiative (IMI) consortium explores opportunities to expedite the development of CAR and TCR engineered T cell therapies in the EU by leveraging tools within the existing EU regulatory framework to facilitate an iterative and adaptive learning approach across different product versions with similar design elements or based on the same platform technology. As understanding of the linkage between product quality attributes, manufacturing processes, clinical efficacy and safety evolves through development and post licensure, opportunities are emerging to streamline regulatory submissions, optimize clinical studies and extrapolate data across product versions reducing the need to perform duplicative studies. It is worth noting that this paper is focusing on CAR- and TCR-engineered T cell therapies but the concepts may be applied more broadly to engineered cell therapy products (e.g., CAR NK cell therapy products).

## Evaluating multiple versions of an engineered T Cell product and process

In the absence of predictive non-clinical models, developers of CAR- and TCR-engineered T cells may need to determine in early clinical development which version(s) of a product should be advanced into later-stage clinical trials. Several engineering options or major manufacturing process alterations may need to be evaluated to arrive at the T cell product candidate with the optimal safety and efficacy profile.

**Figure 1:**
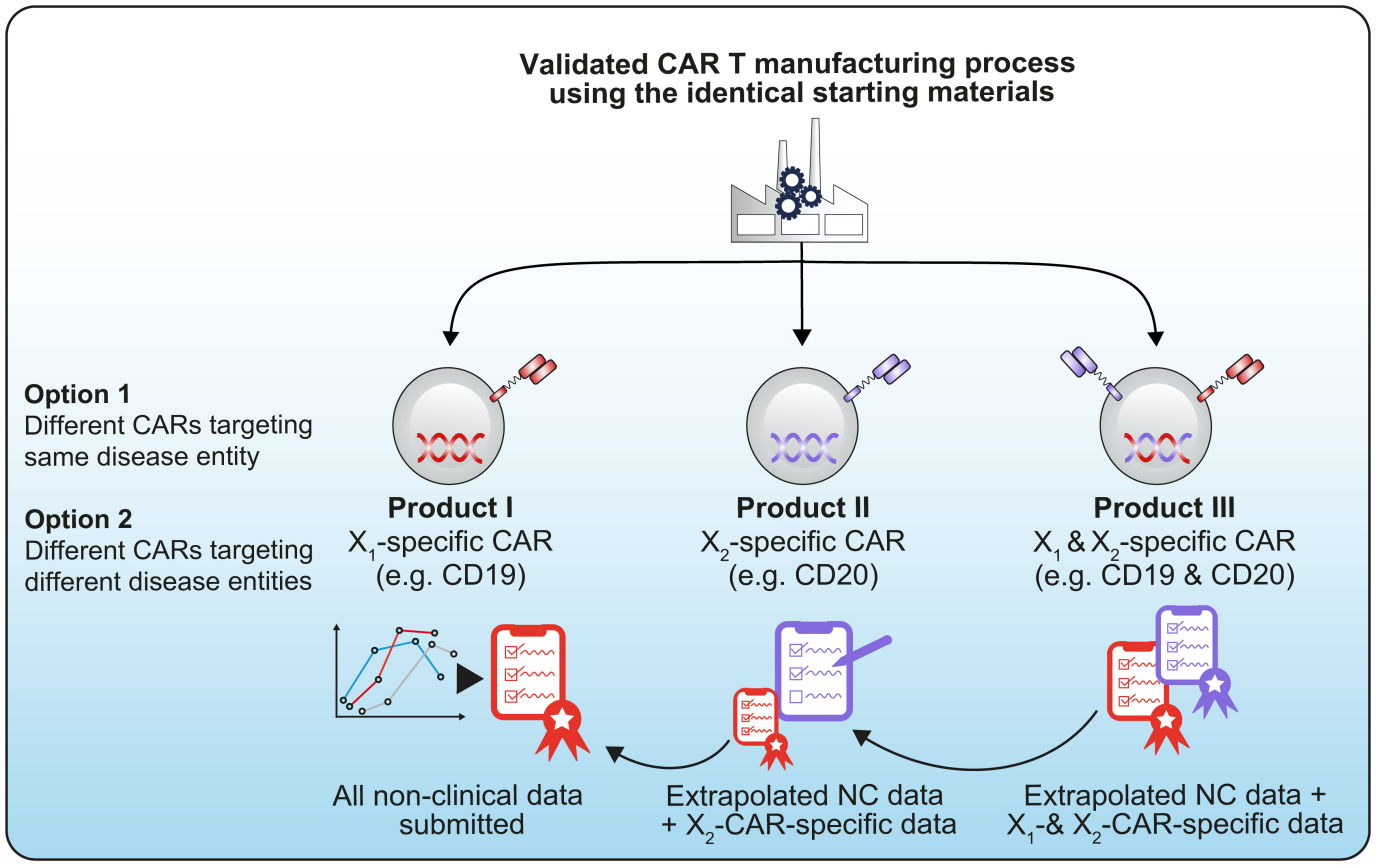
Example of a technology platform using the same starting materials and same manufacturing process before being differentiated into different CAR constructs, for example a CD19/CD20 CAR (1).

To determine the best candidate and/or manufacturing process to progress in later phases of development, developers may test multiple iterations and/or versions of a product that may or may not utilize the same platform technology or manufacturing process. In other circumstances, developers may intend to advance (simultaneously or subsequently) multiple product candidates based on the same platform technology but aimed at treating different targets for the same or different diseases (**Figure 1**).

Several closely related potential product candidates could be most efficiently tested in a single, small, early-phase clinical study. The current framework is reviewed here to explore options for efficient parallel vs. iterative clinical development.

### Current approaches for studying multiple versions of a product

For developers and regulators, being able to investigate several versions of their potential medicines or several products generated from their platform technology in an umbrella trial (a single trial aiming at investigating several investigational medicinal products (IMPs) in a single disease), will save time and resources. Umbrella studies in early phases of development would be aimed at guiding decisions on which version(s) of the product to pursue for further development in later-phase studies. This approach can be especially helpful for developers of cell and gene therapy (CGT) products, where a sponsor may need to evaluate which vector or which exact version of transgene to use, or for platform technology developers exploring different targets. Current regulatory procedures for complex clinical trials can make it difficult and burdensome to manage testing several closely related potential candidates.

**Figure 2:**
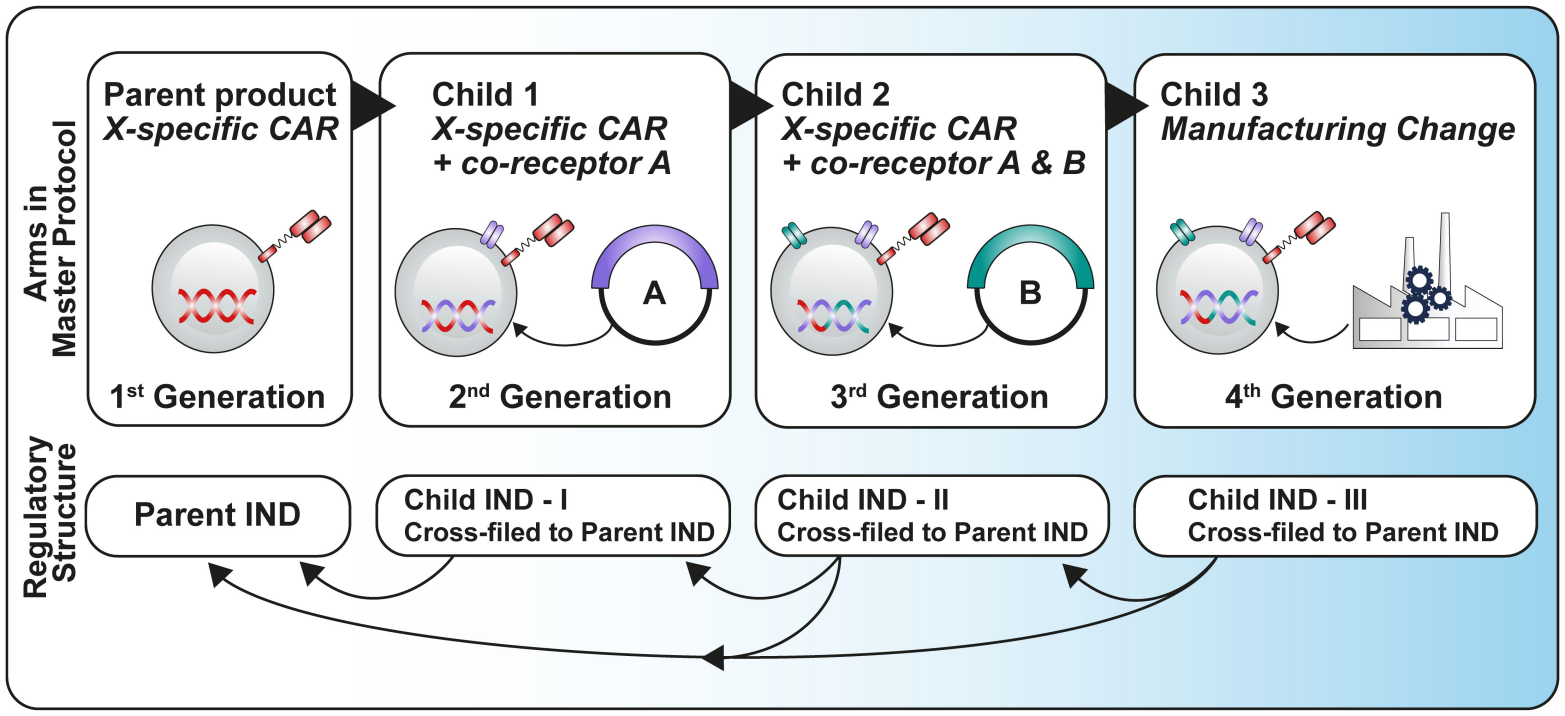
Parent-child approach adapted from Britten et al. (3).

Some publications (2, 3) discuss a more efficient regulatory approach for early development studies by advocating for a ‘parent-child’ approach to Investigational New Drug (IND) submissions, which relies heavily on cross-referencing between a parent IND, containing the master protocol and all common elements of the study and several child INDs containing product-specific details. It is important to note that currently the parent-child IND concept only applies in the US regulatory framework (**Figure 2**).

In 2022, this approach was adopted in the FDA guidance on ‘studying multiple versions of cell and gene therapies’ (4). If a sponsor is using an umbrella trial to study multiple versions of a cellular or gene therapy product for a single disease, the guidance provides recommendations on how sponsors can structure and cross reference INDs to provide all necessary information to the FDA while minimizing redundant submissions of the same information for multiple INDs. However, the studies performed under this guidance are not expected to provide primary evidence of effectiveness to support a marketing authorization application and generally are not adequately powered to demonstrate a statistically significant difference in clinical efficacy between the study arms.

**Figure 3:**
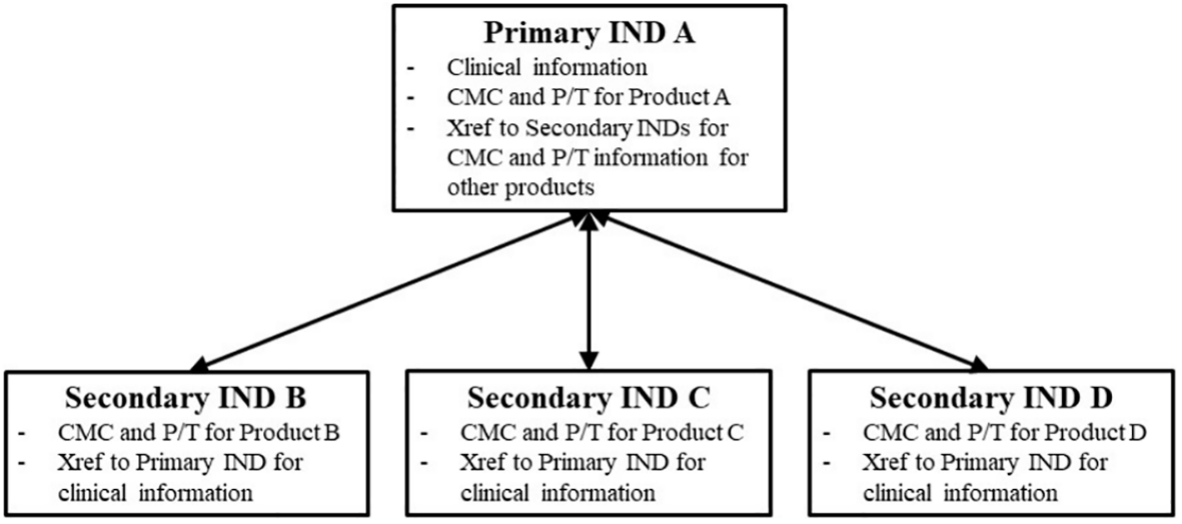
Schematic representation of the primary and secondary IND framework. Source: FDA Guidance for Industry Studying Multiple Versions of a Cellular of Gene Therapy Product in an Early-Phase Clinical Trial (4).

According to the FDA, it is recommended that for a clinical study with two different versions of the investigational product (Product A and Product B), the sponsor still submits two separate INDs, IND A and IND B. One of the INDs (IND A) would be designated as the primary IND and would contain all common clinical information for the umbrella trial. A subsequent secondary IND (IND B) would be able to cross-reference the primary IND for this information, which could be followed by additional secondary INDs (e.g., IND C and IND D). Each IND would still contain specific CMC (Chemical, Manufacturing and Controls) and pharmacological/toxicological data for the respective product version (**Figure 3**).

Potential benefits of such an approach may include the use of synergies in study start-up and management, ability to use a single protocol with a shared control arm and generating data for quicker decisions on the optimal product design. Some considerations may however lead a sponsor to decide against using this approach, including the lack of harmonization globally across regions in case a multi-regional clinical trial is considered, a larger and more complex protocol to manage, and the need to submit a separate protocol to continue development of a selected candidate into a later phase. As noted by Taps (5), these considerations may make this approach more suitable for sponsors seeking to expedite selection of an optimal product candidate rather than to accelerate an investigational product to the market faster.

**Figure 4:**
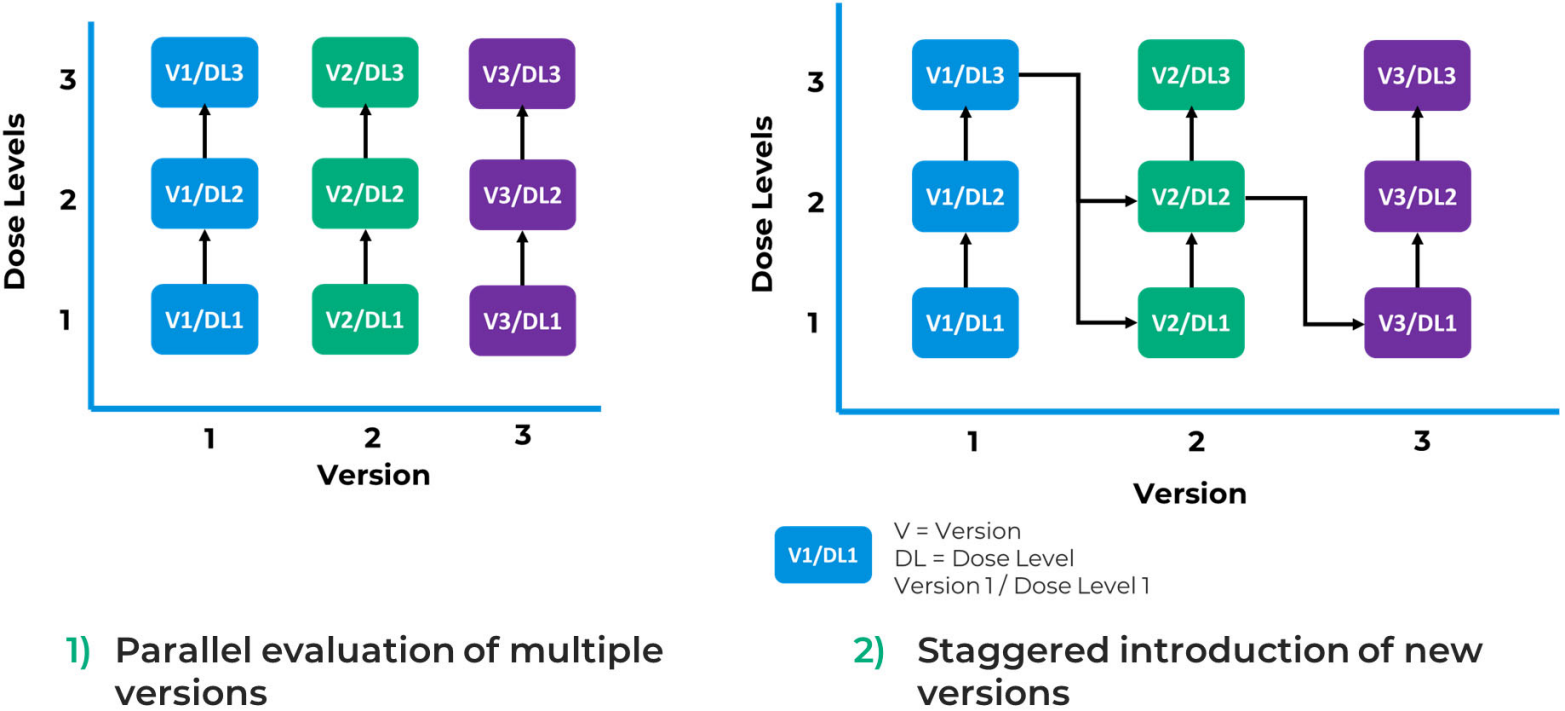
Potential use cases of a parent-child approach.

Taps depicts two potential scenarios to maximize the benefits of this approach (**Figure 4**). First, as mentioned in FDA’s guidance (4), a design aimed at parallel evaluation of multiple versions of a product offers the potential to randomize between arms of the study facilitating comparison of safety and efficacy between versions. However, these comparisons are unlikely to be powered to demonstrate statistically significant differences, particularly where effect sizes between versions are not large. Second, an alternative can be to stagger introduction of a new version in a version escalation approach, where like a traditional dose escalation study, each new version is introduced after some preliminary data are obtained on a prior version. The decision on which data and at what timepoint it would be collected to inform inclusion of an additional version of the product in the study would be pre-specified. Subsequent versions may be more complex or more active than the previous one. This approach offers the opportunity to establish the safety of a less modified or active product version and obtain data that would guide the selection of a potential optimized next generation product.

## Is the FDA’s parent-child approach translatable to the EU?

The approach described in the aforementioned papers (2, 5) and FDA guidance (4) is not directly translatable to the EU context as the regulatory systems for clinical trial authorization in the US and the EU are different. US INDs and EU Clinical Trial Authorization (CTA) applications show some fundamental differences (**Table 1**). INDs are submitted per product and indication and allow for new data and additional clinical study protocols to be accumulated in a single file over time. In contrast, EU CTAs are trial specific and require standalone applications for each new trial. There are also structural differences in how products are assessed by regulators in the EU as compared to the US. In the US, the FDA as a single agency is responsible for regulatory oversight of clinical trials and marketed products. Whereas in the EU, applications for marketing authorization of an ATMP are submitted to European Medicines Agency (EMA) for review on behalf of all Member States but regulatory oversight and assessment of clinical trials is the responsibility of national competent authorities in each EU Member State. The Clinical Trial Regulation has resulted in more harmonization in the scientific assessment of clinical trials, however, depending on the countries participating in a clinical trial, the outcome of the assessment could still be different. For example, when different countries are participating in trial A than trial B the outcome of the assessment could be different even if the trials are using the same or similar compounds. While the IND approach is not applicable in the EU, a more general ‘parent-child’ concept towards product development that allows simultaneous study of different engineering and manufacturing enhancements resulting in a different version of CAR T cell product could be explored in an EU regulatory context.

**Table 1:** Differences between the US and EU system for clinical trials.

IND (US)	CTA (EU)
• Product & Disease/Indication based	• Trial based
• Preclinical data, manufacturing information, detailed clinical protocols and investigator qualifications submitted CTD structure	Content in two parts – 1. Core documents common for all Member States (e.g., Protocol, IB, IMPD, SA, PIP) – single assessment for all 2. Country specific aspects (e.g., ICF, insurance, site details) – Member State assessment
• New information and additional protocols can be added to the IND as product development advances	• A separate CTA is submitted for each new trial with a new/updated IMPD
• Reviewed by single regulatory authority (US FDA)	• Reviewed by competent authorities of each Member State participating in the trial

## What guidance and tools are available in the EU to support a parent-child approach?

### Definition of a new product

One of the important questions to ask when embarking on a parent-child approach to development is whether the specific change to the manufacturing process or engineering enhancement being made to a reference product leads to a new product version or not. FDA’s guidance (4) contains an annex that outlines what the Agency considers to be a new product version vs. unrelated product vs. same product. It is important to point out that while a new product version may have commonalities with its reference product, it could still be considered a distinct product from a regulatory perspective and thus would require a separate marketing authorisation in the end. In the EU, regulators have not elaborated on the concept of different versions of a product.

The EMA published a draft reflection paper (6) providing guidance on the types of differences that could be used to justify a new active substance claim for a genetically modified cell product, and essentially what kind of changes would lead to a new product. A new active substance claim relies on demonstrating that differences are substantial regarding biological characteristics and/or biological activities and/or basic structural elements of the active substance or that the product differs significantly in properties related to safety and/or efficacy.

Justifying a new active substance is a different exercise than demonstrating comparability, where the aim is to show the expected safety and efficacy remains the same/similar after a change is introduced to justify a product should be considered the same as/similar to the reference product (7). EMA’s ‘Toolbox guidance on scientific elements and regulatory tools to support quality data packages for PRIME and certain marketing authorisation applications targeting an unmet medical need, (8) provides some guidance regarding the use of a risk-based approach to tailor a comparability study. These tools are open to interpretation when it comes to translating the parent-child concept to an EU environment. Currently, there is no specific guidance on the types of product versions that could be studied in a parent-child like approach in the EU.

### Master protocol and CTA submission

In the EU, a master protocol can be used for umbrella trials studying multiple versions of a CGT product in a single disease. Managing master protocols in the current EU Clinical Trials Information System (CTIS) can be very complicated for developers and there is some guidance available to assist sponsors who want to make use of this approach.

**Figure 5:**
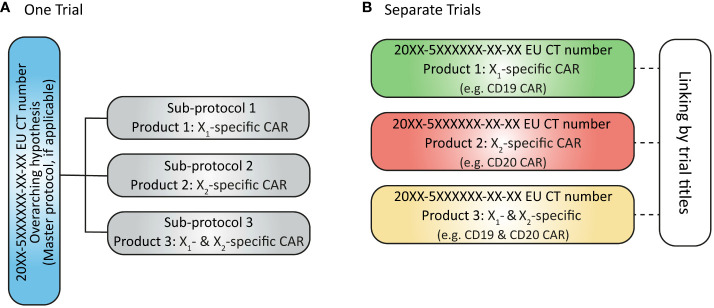
**(A)** Submission of complex clinical trial as one trial with one EU CT number issued; **(B)** Submission of sub-protocols in a complex clinical trial as separate (but linked) trials with separate EU CT numbers issued.

As reported by Clinical Trials Facilitation Group (CTFG), complex clinical trial designs were put in place to increase efficiency by optimizing the use of operational resources and allocation of trial subjects to the most suitable sub-protocol or arm. Two different options are proposed for submitting CTAs for a complex clinical trial with a master protocol: (a) as a single CTA including all sub-protocols or (b) as separate CTAs for each sub-protocol (8) (**Figure 5**). Current guidance (9) encourages sponsors to submit each sub-protocol as a separate application, but this is cumbersome for sponsors to manage and defeats the purpose of having a master protocol representing the design of a single trial.

Regulatory guidance (9–11) provided to date has primarily been aimed towards clinical trials powered to demonstrate a statistically significant difference in efficacy between the study arms to provide primary evidence to support a marketing application. Not all aspects described in these guidelines can be followed for early-phase development programmes and so far, little guidance has been provided in terms of complex clinical trials designs in earlier phases where a parent-child approach may be used.

**Figure 6:**
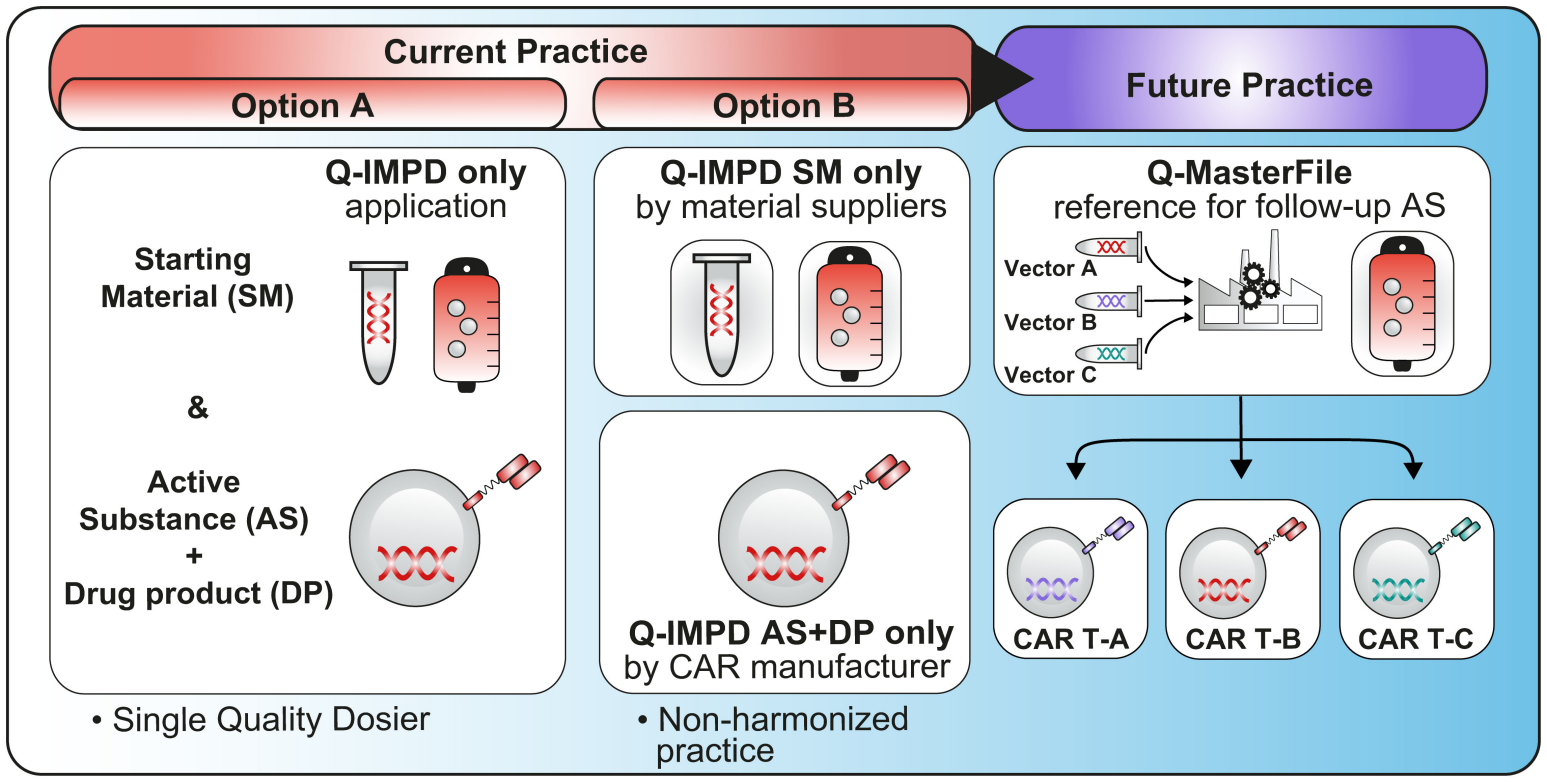
This figure illustrates two currently available options (Option A & B) to use an IMPD-Q only application, either by submitting the entire quality dossier or by submitting a specific part related to the starting material (e.g. supplied by a third party) and a specific part related to the active substance and drug product (Option B would benefit from further guidance to allow a harmonized practice across all NCAs). Finally, this figure illustrates the future practice with the option to have a Q-Master file as described in the new EU regulation.

Other challenges also need to be considered. For example:

Under the new EU CTR, cross-referencing can only be made to a full IMPD. If the child trial involves countries that did not participate in the parent trial, then cross referencing is not possible. In addition, according to Question 2.15 in EU CTR Q&A, v6.5 (July 2023) (12), submission of IMPD-Q to CTIS *via* an initial application for Part I only is possible (“IMPD-Q only application”). Some authorities have already agreed that this option can be used for submission of information on the starting material to ensure that confidential parts are not shared with a third party. In the current framework, this approach would lead to separate submissions for the IMPD-Q only application i.e., IMPD-Q only application for starting material and IMPD-Q only application for the drug product – illustrated in Option B of **Figure 6**. This would be particularly relevant for those developers working with platform technologies.

### Leveraging data across versions

**Figure 7:**
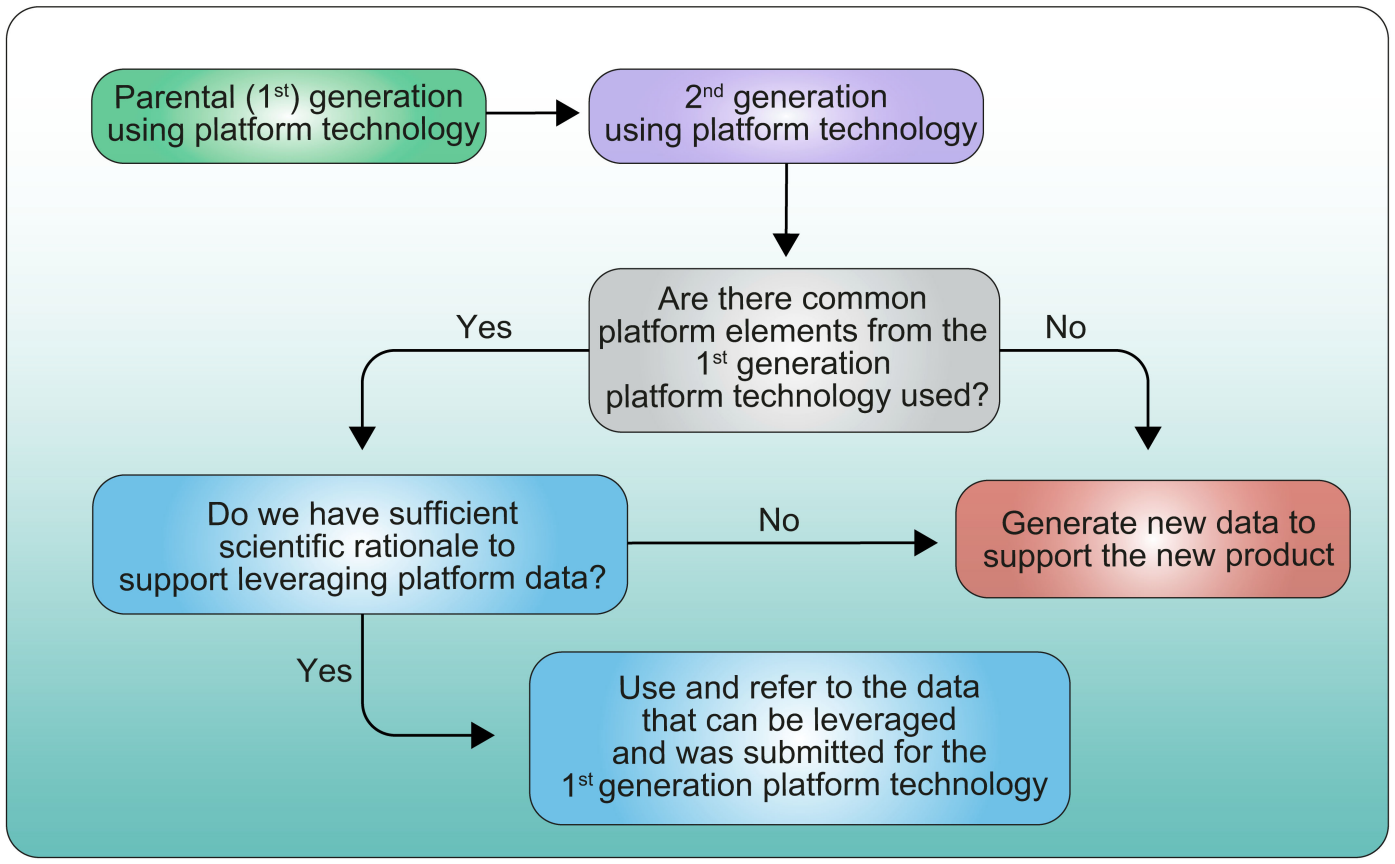
Proposed decision tree for leveraging data from parental generation product to 2^nd^ generation product.

Another aspect of the parent-child approach which is directly linked to the use of platform technologies may be to consider what type of data could be leveraged across product versions to tailor the extent of data to be included in a Clinical Trial Application or the number and type of studies to perform. An example of extrapolated data across the same technology platform has recently been described at the EMA regulatory and scientific virtual conference on RNA-based medicines (13). This could be of particular interest when product versions are being developed sequentially or when more than one version of a product is selected for further development based on early-phase 5studies. As similarly illustrated in **Figure 7** (proposed decision-tree), when product versions are closely related or based on the same platform technology, it should be possible, if scientifically justified, to extrapolate some data from one product version to the next without repeating studies or re-submitting the same information (14).

Justifications/data should be submitted to the Competent Authority to support proposed extrapolation of data as illustrated in **Figure 1**.

Additionally, a white paper on ‘Accelerating The Development of Engineered Cellular Therapies: A Framework for Extrapolating Data Across Related Products’ from Friends of Cancer Research (14) proposes a risk-based approach for extrapolating non-clinical, clinical and CMC data across related product versions.

A concept of the ‘risk-based’ approach for ATMPs has already been introduced in EU legislation (15) and elaborated in EMA guidance (16). The risk-based approach provides an optional strategy for determining the extent of quality, non-clinical and clinical data to be included in a marketing authorisation application (MAA). EMA’s draft guideline on quality, non-clinical and clinical aspects for investigational ATMPs (17) also suggests the risk-based approach may be used to determine the content of an IMPD for clinical trials. The content of an IMPD may be adapted to the risks identified at the beginning of development based on existing knowledge on the type of product and its intended use. This suggests prior knowledge, including that from a previous version of a product, may be used.

Prior knowledge is also identified in the toolbox guidance for PRIME and other applications targeting an unmet medical need (8) as a potential scientific tool that can be used in determining quality data packages. This guidance also notes “platforms” such as similar manufacturing processes and/or analytical tests used across many different products within a group, including genetically modified cell therapies, can generate prior knowledge.

As highlighted in the presentation given during the recent EMA regulatory and scientific virtual conference on RNA-based medicines (13), registration of COVID-19 medicines during the pandemic have opened doors for more flexibilities which could lead to acceleration of the development of innovative medicines. This example demonstrated that it is possible to avoid repetition of unnecessary non-clinical tests for subsequent medicines being developed using the same platform technology. This highlights the importance of looking at lessons learned, and experiences gained during the COVID-19 pandemic to develop mechanisms and guidelines allowing more flexibility when developing such medicines using very complex technologies.

### Scientific advice and dialogue

Due to the complexity and potential methodology considerations involved in a parent-child approach, it is best to seek early advice from EU regulators to ensure the data generated in any studies are sufficient for regulatory purposes. The EMA and national competent authorities provide many opportunities for developers to seek advice (18).

National competent authorities provide scientific and/or regulatory advice, which is particularly useful if a developer is undertaking a study in one or a limited number of countries. The second phase of a pilot for Simultaneous National Scientific Advice (SNSA) is also currently running (19). The pilot aims to create a more efficient process for developers seeking advice from multiple National Competent Authorities (NCAs) on the same questions. However, one of the caveats is that no more than two NCAs could be consulted simultaneously and a third one acting only as observer. EMA also offers centrally coordinated scientific advice procedures to respond to specific questions about the development of a particular medicine (18). EMA scientific advice is typically sought prior to pivotal clinical trials where the data are expected to form the basis for a marketing authorization application. It is worth noting that EMA does not advise on which data are needed to obtain approval for a trial as opposed to National Scientific advice. The EMA Innovation Task Force (ITF) and national innovation offices provide an early entry point for a more general discussion on emerging therapies and technologies. However, these meetings are aimed for products that are still very early in the development and only offers opportunities for general discussions on regulatory pathways for approval of clinical trials. Indeed, as part of those interactions, developers will be given opportunities for brainstorming discussions on general aspects with regulators and national experts.

Several activities are also foreseen to reinforce coordination and facilitate clinical trials through the Accelerating Clinical Trials in the EU (ACT EU) initiative (20). The aim will be to ensure consistency between the different bodies providing scientific advice throughout the lifecycle of a product.

## What additional tools are currently being developed?

### Reflection paper on platform trials

A parent-child approach may involve a platform approach. EMA’s Methodology Working Party is working on a Reflection Paper on Platform Trials that it intends to release for public consultation in March 2024 (21). The paper will aim to clarify terminology and key concepts; describe key methodological aspects unique to platform trials and important design features to help guide planning and protocol development; and outline the Committee for Medicinal products for Human Use (CHMP) position on the increased complexity and uncertainty resulting in platform trials for confirmatory evidence generation.

### ACT-EU – a multi-stakeholder platform

ACT EU is setting up a multi-stakeholder discussion platform (22) aimed at identifying relevant scientific, methodological and technological advances to develop the clinical trial environment in the EU. This forum could provide an opportunity to raise current challenges encountered by developers of CAR T and engineered T cell products and could propose efficient ways to handle multiple versions of a same products or follow-on products developed from a same platform technology. Authorities are expected to release a roadmap with all currently applicable guidance relevant to methodology (inc. complex clinical trials) and any planned guidance for development together with stakeholders, including Sponsors. A workshop specifically focusing on methodology will also be organized.

### Proposed revision to EU legislation

In April 2023 the European Commission proposed a revision of EU pharmaceuticals legislation (23). Included in the proposed changes is the potential for additional quality master files that could address the current lack of options in the EU to cross-reference starting materials. However, it is still unclear whether this proposal will be adopted, and it will likely take several years before it can be implemented.

## Case-study: introduction of a 2^nd^ generation T cell product in an ongoing first-in-human clinical trial

Dr. Miriam Meyer (Immatics Biotechnologies GmbH, Tübingen) presented a successful case study at a recent conference organized by the German Society of Gene Therapy and the Paul Ehrlich Institute (PEI) on the 27^th^ of April 2023 in Langen, Germany (24). The presentation showcased the investigation of two versions of a TCR T cell therapy in a FIH clinical trial.

The clinical trial presented was ACTengine^®^ IMA203-101 (25), a Phase 1/2 study evaluating genetically modified autologous T cells expressing a T cell receptor (TCR) recognizing a cancer/germline antigen as monotherapy or in combination with nivolumab in patients with recurrent and/or refractory solid tumors. The study is conducted in the US as well as in Germany (under the Clinical Trial Directive 2001/20/EC which has been replaced by the Clinical Trial Regulation EU 536/2014 (CTR)).

The clinical trial design was initially set up to investigate IMA203, an autologous CD8^+^ T cell product candidate for intravenous (i.v.) infusion derived from cancer patients’ own peripheral blood and engineered to express a TCR specific for an HLA-A*02:01-restricted peptide derived from PRAME (preferentially expressed antigen in melanoma). IMA203 was developed based on Immatics’ target and TCR discovery platforms XPRESIDENT^®^ and XCEPTOR^®^. At this stage, the clinical trial design was composed of a IMA203 dose escalation part, a dose expansion part at the recommended phase II dose, and a cohort for combination of IMA203 with the checkpoint inhibitor nivolumab.

Encouraged by a well manageable tolerability profile and first signs of anti-tumor activity during dose escalation with the 1st generation product candidate, Immatics decided to introduce a potency-enhanced 2nd generation TCR T approach called IMA203CD8 in a separate cohort in the ongoing IMA203-101 trial and leverage data from the 1st generation product as well as existing operational processes to accelerate clinical evaluation of the 2nd generation asset.

To implement the new 2nd generation cohort in the IMA203-101 clinical trial protocol, the primary-secondary IND approach has been employed by Immatics according to FDA Industry guidance on “Studying Multiple Versions of a Cellular or Gene Therapy Product in an Early-Phase Clinical Trial”, Nov 2022. The IND for the IMA203 1st generation product candidate was assigned the primary IND. An amended clinical trial protocol introducing the 2nd generation product candidate IMA203CD8 with an abbreviated dose escalation scheme and a dose expansion cohort was submitted to the primary IND. In parallel, a submission for a new IND assigned secondary IND for the 2nd generation product candidate IMA203CD8 was performed. This secondary IND comprised all information specific to IMA203CD8.

In Germany, Immatics presented the plans for the introduction of the 2nd generation product candidate IMA203CD8 to the PEI for a written advice and aligned with the agency that the updated clinical trial protocol and documentation specific to this asset could be submitted to the already approved CTA for IMA203-101 as a substantial amendment.

This example shows that - at least under the former Clinical Trial Directive - the European regulatory system allowed the flexibility that is required to study different versions of a cellular therapy in one trial.

During the panel discussion with regulators at the German gene therapy/PEI theme day “The next frontiers in ATMP development” on 27^th^ of April 2023 in Langen it was emphasized by regulators that this flexibility would still be possible in the EU under CTR.

## What are the T2EVOLVE recommendations?

Currently, there is a lack of EU guidance and mechanisms on how to efficiently handle early clinical trials aimed at selecting the version(s) of the product to be pursued for later-stage of the development and how to efficiently handle CTAs based on platform technology.

T2EVOLVE would like to provide the following recommendations:

### Workshops and Q&As

Guidance for complex clinical trials is designed for products that are intended to be marketed. Currently, there are no guidelines for early-phase development trials. Cell and gene therapies are rapidly evolving, whereas development of new guidelines can be very lengthy and by the time guidelines are developed science has evolved and some of the aspects may no longer be applicable. Additionally, during the pandemic we have gained experience in efficiently handling innovative technologies. It is important that lessons learned from the products approved during the pandemic are considered to adapt our current guidance based on experience. Therefore, developers would welcome dialogue focused on specific solutions for cell and gene therapies in the form of workshops/Q&As with more concrete examples between developers and regulators perhaps under the umbrella of ACT EU.

### Scientific advice

Lack of specific guidance could be mitigated by scientific advice between regulators and developers to discuss study design and get a harmonized view across the different NCAs. Regulators encourage early dialogues to discuss complex clinical trials as discussed above. However, developers would benefit from having a multi-disciplinary discussion platform at different stages of the development to ensure that innovative clinical trials are being assessed properly and that the best options/route are selected by the sponsors. It would be important that all National Competent Authorities participate to these dialogues since EMA is not responsible for approval of Clinical Trial Applications.

At the moment, although the SNSA pilot allows simultaneous scientific advice involving two countries and one country observer, there is still a lack of forum for which this type of clinical study could be openly discussed in more detail with regulators allowing developers to get a consolidated advice across EU countries. Indeed, T2EVOLVE would welcome a similar program as the Complex Innovative Trial Design (CID) meeting program put in place by the FDA (26). This pilot gives the opportunity to developers to meet with FDA (face-to-face or virtually) multiple times to have a multidisciplinary discussion to assess the proposed innovative clinical trial design and ensure that the best possible option for testing a specific drug is selected.

ACT EU is encouraged to address this need as the initiative establishes the multi-stakeholder platform to facilitate dialogue between clinical trial stakeholders, including patients, healthcare professionals and academia.

### Guidelines on the following aspects would be welcomed

Regarding the case study related to IMA203 described above, the flexibility from PEI in allowing a substantial amendment to include a different version of a product in the same clinical trial was very welcomed. However, it is unclear whether this flexibility will be accepted by all Member States and therefore, T2EVOLVE would highly welcome EU guidance on this matter so that this approach can be applicable and harmonized across all EU competent authorities and within CTIS.

As described in this paper, various tools are already available in the EU. However, those tools cannot always be translated to the development of different versions of the same products at an early stage of the development or when multiple products are being developed from the same platform technology. It would be helpful for developers to have consolidated recommendations provided in a specific guideline. Indeed, having more specific guidelines would guide less experienced authorities/developers on this approach thereby fostering innovation and faster patient access in the EU.

Such guidelines would cover for example:

- Clearer guidance on what is defined as a new product version vs. unrelated product vs. same product and on how to extrapolate data between different versions.
- For companies working on platform technology, guidance on what could be considered a platform technology and how to justify cross-reference of certain sections of the dossier would be necessary to ensure efficiency. Some guidelines refer to risk-based approach, but further guidance on platform technologies is necessary to help companies leveraging scientific knowledge from a single platform technology and ensuring that an adequate risk-based approach is performed to avoid repeated testing (for instance toxicological studies) (11).
- An updated version of initiation and conduct of complex clinical trial guidance relating to CTR and CTIS implementation is needed. As CTIS is working on CTA level, a guidance for cross-referencing/cross-linking to master protocol and practical implementation through CTIS improvements would be welcomed. For regulators, having a mechanism that would ensure that sections of the dossiers/CTA already reviewed and approved could easily be retrieved would be largely beneficial to avoid duplication of work and ensure that knowledge related to a specific drug or platform technology is transferred adequately across the different countries/assessors.

Further improvement to the EU CTR and CTIS is needed to ensure greater efficiency. An interesting proposal would be to consider having all Part 1 applications including all documentation and assessment reports available to all Member States irrespective of whether they are participating in a specific Clinical Trial. This would give the option to refer to already submitted data even though, it would not eliminate the need for Member States who have not participated in the previous trial, to assess the newly submitted data to them and make their own decision on whether the data could be accepted from their point of view.

### Master file or IMPD master protocol

The use of a master file or IMPD master manufacturing protocol linked to a clinical trial (like what is done for conventional ASMF) can potentially match ideas from improved parent-child approach. IMPD-Q master manufacturing protocol will contain elements subject to standardization and will reflect part of the production that is less subjected to modifications. If adopted, the new EU legislation would allow the option to file additional quality master files and would help companies to handle complex platform technology in a more efficient and agile manner but it is unclear when and how this would be made available to companies (future practice of **Figure 6**).

In the meantime, and as described in **Figure 6**, developers can use the IMPD-Q only application (Option A – **Figure 6**). However, at the moment there are no harmonized views as to whether all Member States would accept to have a separate submission of confidential sections (Option B - **Figure 6**) such as for instance starting materials supplied by a third party.

Currently, developers would need to consult with National Competent Authority to get some clarifications on how to handle IMPD-Q applications to allow confidential information to be submitted in CTIS. With the objective that improvements are made to CTIS in the long run, T2EVOLVE would expect that further guidance will be provided by EMA in the future. However, if we consider the potential Q-Master file option as described in the new EU legislation, we could wonder whether CTIS would be able to support multiple Q-Master Files to address confidentiality aspects.

## Conclusion

As described in this paper, the parent-child approach as proposed by the FDA is not directly translatable to the EU regulatory framework. Multiple tools/guidelines are already available but there are no specific guidelines for developing several versions of a product at an early phase of a clinical trial or developing several versions aiming at targeting a single patient/single disease or multiple patients/multiple diseases derived from the same platform technology.

Indeed, it was reported that EU is not very attractive when it comes to the choice of opening clinical trials compared to the US or Asia which is further reflected in the recent ARM report Regenerative Medicine: The Pipeline Momentum Builds (27). It shows that US and Asia are leading in active ATMP clinical trials with EU taking the smallest share of new trials. Helping developers to navigate this complex EU regulatory framework with agility when developing innovative technologies will lead to greater efficiencies and be a step forward to make EU more attractive.

T2EVOLVE is therefore advocating to have a clear mechanism and specific guidelines that would aim at helping developers to accelerate the development of innovative cellular therapy products. This would be in line with the current objective set by ACT EU (25) and would aid in attracting new clinical trials in the EU.

## Glossary and definitions

### Platform technology

Article 8 of the new Directive indicates that a platform technology is ‘a medicinal product comprised of a fixed component and a variable component that is pre-defined in order to, where appropriate, target different variants of an infectious agent or where necessary to tailor the medicinal product to characteristics of an individual patient or a group of patients (‘platform technology’)’ (article 15(2) of revised Directive proposed by European Commission on 26 April 2023 (23).

### Parent-child concept

As described by Stewart et al. (2020), Parent IND or CTA would contain common sections providing all relevant information for the candidates or manufacturing alterations (2). Each child IND or CTA would cross-reference common sections while providing only the candidate- or process-specific information.

### Umbrella trial

A trial designed to investigate the safety/efficacy/effects of several IMPs in a single population with the same disease (9).

### Platform trial

A type of complex clinical trial characterized by a shared operational framework that allows for the investigation of multiple IMPs in a continuous manner, possibly in different diseases/conditions, with different IMPs ‘entering’ and ‘leaving’ the platform at different times based on pre-specified decision rules (10).

### Master protocol

A typical master protocol describes the overall clinical trial design including components and operational aspects applicable to all related sub-protocols such as the clinical trial rational, objectives, endpoints, benefit-risk assessment, shared procedures regarding safety monitoring and reporting, and a common screening platform dictating trial subject eligibility and/or treatment allocation. The master protocol should clearly describe how trial subjects are allocated to the individual sub-protocols or arms and should describe decision criteria for opening and closing of sub-protocols/arms as well as for re-allocating trial subjects from one sub-protocol to another, if applicable. Master protocols are often applied to particular study designs such as basket, umbrella, or platform designs (10).

## Author contributions

DA: Conceptualization, Writing – original draft, Writing – review & editing. IS: Conceptualization, Writing – original draft, Writing – review & editing. ML: Visualization. MH, Writing - review & editing: Writing – original draft. MM: Writing – original draft. TT: Writing – original draft. BS: Writing – original draft, Writing – review & editing. ZI: Writing – review & editing. CS: Writing – original draft. PF: Conceptualization, Writing – original draft, Writing – review & editing. UK: Writing – original draft. HN: Conceptualization, Writing – original draft, Writing – review & editing. IJ: Writing – original draft. JA-C: Conceptualization, Writing – original draft, Writing – review & editing.

## References

[1] Tong C Zhang Y Liu Y Ji X Zhang W Guo Y. Optimized tandem CD19/CD20 CAR-engineered T cells in refractory/relapsed B-cell lymphoma. Blood (2020) 136(14):1632–44. doi: 10.1182/blood.2020005278 PMC7596761 32556247

[2] Stewart M Keane A Butterfield L Levine B Thompson B Xu Y. Accelerating the development of innovative cellular therapy products for the treatment of cancer. Cytotherapy (2020) 22(5):239–46. doi: 10.1016/j.jcyt.2020.01.014 32199724

[3] Britten CM Shalabi A Hoos A. Industrializing engineered autologous T cells as medicines for solid tumours. Nat Rev Drug Discovery (2021) 20(6):476–88. doi: 10.1038/s41573-021-00175-8 33833444

[4] US Food and Drug Administration. Studying Multiple Versions of a Cellular of Gene Therapy Product in an Early-Phase Clinical Trial, Guidance for Industry (2022). Available at: https://www.fda.gov/regulatory-information/search-fda-guidance-documents/studying-multiple-versions-cellular-or-gene-therapy-product-early-phase-clinical-trial (Accessed 1 Aug 2023).

[5] Taps T. The parent–child IND approach: an interpretation of FDA’s guidance on studying multiple versions of a cellular or gene therapy product in an early-phase clinical trial. Cell Gene Ther Insights (2022) 8(11):1581–6. doi: 10.18609/cgti.2022.229

[6] European Medicines Agency. Criteria to be considered for the evaluation of new active substance (NAS) status of biological substances. Available at: https://www.ema.europa.eu/en/criteria-be-considered-evaluation-new-active-substance-nas-status-biological-substances (Accessed 1 Aug 2023).

[7] European Medicinies Agency. Questions and answers Comparability considerations for Advanced Therapy Medicinal Products (ATMP). Available at: https://www.ema.europa.eu/en/documents/other/questions-answers-comparability-considerations-advanced-therapy-medicinal-products-atmp_en.pdf (Accessed 1 Aug 2023).

[8] European Medicines Agency. Toolbox guidance on scientific elements and regulatory tools to support quality data packages for PRIME and certain marketing authorisation applications targeting an unmet medical need. Available at: https://www.ema.europa.eu/en/toolbox-guidance-scientific-elements-regulatory-tools-support-quality-data-packages-prime-certain (Accessed 1 Aug 2023).

[9] Clinical Trials Facilitation and Coordination Group (CTFG). Recommendation Paper on the Initiation and Conduct of Complex Clinical Trials (2019). Available at: https://www.hma.eu/fileadmin/dateien/Human_Medicines/01-About_HMA/Working_Groups/CTFG/2019_02_CTFG_Recommendation_paper_on_Complex_Clinical_Trials.pdf (Accessed 1 Aug 2023).

[10] ACT-EU. Complex clinical trials – Questions and answers (2022). Available at: https://health.ec.europa.eu/system/files/2022-06/medicinal_qa_complex_clinical-trials_en.pdf (Accessed 1 Aug 2023).

[11] CTCG Q&A on Submission of Complex Clinical Trials in CTIS, v 1.0, 14 (2023). Available at: www.hma.eu/fileadmin/dateien/HMA_joint/00-_About_HMA/03-Working_Groups/CTCG/2023_03_CTCG_QA_complex_clinical_trials_and_CTIS_v1.0.xlsx (Accessed 1 Aug 2023).

[12] EudraLex. Clinical trials guidelines - Questions and Answers Document - Regulation (EU) 536/2014 – Version 6.5 (2023). Available at: https://health.ec.europa.eu/system/files/2023-07/regulation5362014_qa_en.pdf (Accessed 1 Aug 2023).

[13] Goody S. Application of RNA technologies: non-clinical industry perspective, in: EMA Regulatory and scientific virtual conference on RNA-based medicines (2023). Available at: https://www.ema.europa.eu/en/documents/presentation/presentation-application-rna-technologies-non-clinical-industry-perspective-susan-goody_en.pdf (Accessed 1 Aug 2023).

[14] Friends of Cancer Research. Accelerating The Development of Engineered Cellular Therapies: A Framework for Extrapolating Data Across Related Products (2023). Available at: https://friendsofcancerresearch.org/wp-content/uploads/Accelerating_The_Development_of_Engineered_Cellular_Therapies.pdf (Accessed 1 Aug 2023).

[15] Annex I. Part IV of Directive 2001/83/EC of the European Parliament and of the Council of 6 November 2001 on the Community code relating to medicinal products for human use. Available at: https://eur-lex.europa.eu/legal-content/EN/TXT/?uri=CELEX%3A02001L0083-20220101 (Accessed 1 Aug 2023).

[16] European Medicines Agency. Guideline on the risk-based approach according to Annex I, part IV of Directive 2001/83/EC applied to Advanced Therapy Medicinal Products (2013). Available at: https://www.ema.europa.eu/en/documents/scientific-guideline/guideline-risk-based-approach-according-annex-i-part-iv-directive-2001/83/ec-applied-advanced-therapy-medicinal-products_en.pdf (Accessed 1 Aug 2023).

[17] European Medicines Agency. Guideline on quality, non-clinical and clinical aspects for investigational ATMPs (2019). Available at: https://www.ema.europa.eu/en/guideline-quality-non-clinical-clinical-requirements-investigational-advanced-therapy-medicinal (Accessed 1 Aug 2023).

[18] European Medicines Agency. Scientific Advice on medicines for Human use in the EU medicines regulatory network (2022). Available at: https://www.ema.europa.eu/en/documents/other/science-advice-medicines-human-use-eu-medicines-regulatory-network_en.pdf (Accessed 1 Aug 2023).

[19] Heads of Medicines Agencies and European Medicine Agency. Guidance for applicants on Simultaneous National Scientific Advice (SNSA) phase 2 pilot (from October 2022) – Optimized process (europa.eu) (2022). Available at: https://www.ema.europa.eu/en/documents/other/guidance-applicants-simultaneous-national-scientific-advice-snsa-phase-2-pilot-october-2022_en.pdf (Accessed 1 Aug 2023).

[20] European Medicines Agency. Accelerating Clinical Trials in the EU (ACT EU) initiative (2023). Available at: https://www.ema.europa.eu/en/human-regulatory/research-development/clinical-trials/accelerating-clinical-trials-eu-act-eu (Accessed 1 Aug 2023).

[21] European Medicines Agency. Concept paper on platform trials. Available at: https://www.ema.europa.eu/en/documents/scientific-guideline/concept-paper-platform-trials_en.pdf (Accessed 1 Aug 2023).

[22] ACT EU. Priority Action 3 concept paper: an EU multi-stakeholder platform for improving clinical trials (2023). Available at: https://www.ema.europa.eu/en/documents/scientific-guideline/priority-action-3-concept-paper-eu-multi-stakeholder-platform-improving-clinical-trials-accelerating_en.pdf (Accessed 1 Aug 2023).

[23] European Commission. Reform of the EU pharmaceutical legislation (2023). Available at: https://health.ec.europa.eu/medicinal-products/pharmaceutical-strategy-europe/reform-eu-pharmaceutical-legislation_en (Accessed 1 Aug 2023).

[24] Die Deutsche Gesellschaft für Gentherapie e.V. (DG-GT). The Next Frontiers in ATMP Development. Available at: https://www.dg-gt.de/event-home (Accessed 1 Aug 2023).

[25] ClinicalTrials.gov. ACTengine® IMA203/IMA203CD8 as Monotherapy or in Combination With Nivolumab in Recurrent and/or Refractory Solid Tumors (ACTengine). Available at: https://classic.clinicaltrials.gov/ct2/show/NCT03686124 (Accessed 1 Aug 2023).

[26] US Food and Drug Administration. Interacting with the FDA on Complex Innovative Trial Designs for Drugs and Biological Products Guidance for Industry (2020). Available at: https://www.fda.gov/regulatory-information/search-fda-guidance-documents/interacting-fda-complex-innovative-trial-designs-drugs-and-biological-products (Accessed 1 Aug 2023).

[27] Alliance for Regenerative Medicine. Regenerative Medicine: The Pipeline Momentum Builds (2022). Available at: https://alliancerm.org/sector-report/h1-2022-report/ (Accessed 1 Aug 2023).

